# When Coproduction Is Unproductive: Comment on "Experience of Health Leadership in Partnering with University-Based Researchers in Canada: A Call to ‘Re-Imagine’ Research"

**DOI:** 10.15171/ijhpm.2019.140

**Published:** 2019-12-22

**Authors:** Sara A. Kreindler

**Affiliations:** Department of Community Health Sciences, Faculty of Health Sciences, University of Manitoba, Winnipeg, MB, Canada.

**Keywords:** Research Partnerships, Integrated Knowledge Translation, Health Systems, Health System Leadership

## Abstract

Bowen et al offer a sobering look at the reality of research partnerships from the decision-maker perspective. Health leaders who had actively engaged in such partnerships continued to describe research as irrelevant and unhelpful – just the problem that partnered research was intended to solve. This commentary further examines the many barriers that impede researchers from meeting decision-makers’ knowledge needs, and decision-makers from using knowledge that they have coproduced. It argues that not all barriers can or should be dismantled: some are legitimate and beneficial; some are harmful but deeply entrenched; some arise unpredictably. This being the case, it seems unrealistic to expect either existing or emerging strategies to create a macro-context devoid of barriers to the fruitful coproduction of knowledge. However, it may be possible to identify and support micro-contexts (configurations of participants, settings, and project characteristics) in which partnered research is most likely to achieve its aims.

Bowen et al provide an important reality check about researcher–decision-maker coproduction of knowledge, also known as partnered research or integrated knowledge translation (IKT).^1^ Coproduction has been advanced as a solution to the “knowledge production problem” – namely, the dearth of research relevant to the real-world questions with which decision-makers engage – held to underlie decision-makers’ non-use of research evidence.^2^ Yet despite strenuous efforts by multiple parties (including funders) to promote coproduction, the problem apparently persists: The decision-makers in Bowen et al’s study, all of whom had taken “an active leadership role in research partnerships,” nonetheless reported that research continued to be “experienced as unhelpful or irrelevant to decision-making.”

These findings remind us that decision-maker involvement in research is not a panacea; nor is it an end in itself – it is a means toward ensuring that decision-makers have the knowledge they need to improve healthcare. Even when coproduction is undertaken, barriers remain that impede this enterprise from generating knowledge that is useful and/or used. The better the barriers are understood, the greater our prospect of creating conditions for success.

When examining barriers, we should not assume that all can and should be dismantled. Some barriers are legitimate or beneficial in some way; some are indeed deleterious and removable; some cannot be targeted for removal because they spring up unpredictably. This distinction between the good, the bad, and the unavoidable is equally applicable to barriers that arise on the researcher and decision-maker sides of a partnership. Put differently, there are good, bad, and unavoidable reasons for researchers’ failure to provide the knowledge decision-makers need, and for decision-makers’ failure to use knowledge for which they have expressed a need.

This study, which highlighted decision-maker perspectives, uncovered a plethora of bad reasons for researchers’ unwillingness or inability to meet decision-makers’ knowledge needs. These include unfortunate characteristics of the researcher (eg, lack of respect, understanding, or willingness to venture beyond a narrowly defined specialty) or of the research context (eg, disproportionate ethics-review processes that impose long delays on minimal-risk research, academic reward structures that discourage risk-taking, hurry-up-and-wait funding cycles that distort project timelines) that scuttle meaningful collaboration.^1^ However, at least one good reason should also be recognized: When the knowledge that decision-makers require is not *research* knowledge, it is legitimate for researchers to decline involvement in its acquisition. Notwithstanding the desirability of eliminating arbitrary distinctions between official and unofficial (ie, quality improvement or evaluation) research, the distinction between research and non-research is not in itself arbitrary. A defining quality of research is the generation of findings of relevance beyond the immediate situation (ie, generalizable or transferable).^3^ Some organization-specific investigations may not meet this criterion; others may technically meet it but lack sufficient novelty to advance the field.^4^ This observation is not meant to diminish the value of such investigations to the requesting organization – it may, for example, be important to know which of many well-studied factors is most responsible for a particular local problem, or to confirm that a gold-standard intervention has been implemented with fidelity and is achieving the expected outcomes. However, projects of this ilk serve a different social good than that which it is the university’s mandate to pursue. It would seem reasonable to expect organizations to address non-research questions using their own resources, not the university’s (beyond what university-based researchers may choose to offer in the interests of relationship-building). The “unavoidable” category includes such reasons as research proving unusable due to its inconclusiveness, or to a need for additional advances in knowledge before it can be used to generate recommendations (and researchers might be forgiven for declining to help decision-makers “jump to solutions” for a poorly understood problem). Meanwhile, some research questions cannot be answered without an unacceptable outlay of time and money, or perhaps at all (as when necessary pre-intervention data are of abysmal quality or were never collected).

Shifting now to a consideration of barriers on the decision-maker side, proponents of IKT have long recognized at least one good reason why decision-makers may not act upon knowledge that they requested: Multiple legitimate influences impinge on decision-making; researchers cannot expect their findings to be the sole deciding factor.^5^ Tight decision-making timelines are sometimes characterized as a good reason why decision-makers cannot wait for research findings, although this characterization seems open to question: In one documented case, a health system made repeated, unsuccessful efforts, spanning more than a decade, to solve one particular problem; each effort was marked by a flurry of panicked activity and an insistence that there was no time for further information-gathering or deliberation.^6^ On this note, there are many bad reasons – some mentioned in this article, others elsewhere. For example, decision-makers may request evidence on a decision so highly politicized, or under the control of individuals with such rigid opinions, that the “voice” of research is sure to be silenced. Other bad reasons reflect pervasive features of the organizational context that prevent knowledge from being incorporated into decision-making; in particular, an organization may lack *absorptive capacity* , the capacity to acquire, assimilate, and apply new knowledge (of any kind, not limited to research evidence).^7^ All three components of this tripartite capacity are essential: If an organization does not value or support knowledge acquisition, it is highly unlikely to nurture research partnerships; if it does not afford managers the time or permission to assimilate new ideas, relevant findings may fail to be recognized as useful; if its decision-making style consists of crisis-driven “idea imposition,” then even knowledge that managers have assimilated may be ignored when the time comes to make an actual decision.^8^ Such organizational dysfunction may also impair the coproduction of knowledge by distorting the questions that decision-makers ask: those who inhabit a poor decision-making environment may be motivated to request knowledge they will not or cannot apply, merely to create an appearance of addressing the issue (*symbolic use*),^9^ or may be unable to discern what knowledge they need. Finally, there are unavoidable reasons such as decision-maker turnover, organizational restructuring, and unforeseen developments that alter organizational priorities.

Given the number and diversity of barriers to fruitful partnered research, it should not be surprising when mechanisms for promoting it prove limited in their effectiveness. In particular, this study draws attention to the limitations of funding opportunities designed to incentivize IKT.^1^ Such funding schemes are blunt instruments, addressing certain barriers on the researcher side (notably, lack of resources or academic recognition for IKT) but leaving others – as well as the many barriers on the decision-maker side – untouched. They are vulnerable to gaming by researchers who engage decision-makers tokenistically, and may produce unintended consequences (eg, organizations may become inundated with researcher demands for support of investigator-driven projects that pose hidden costs but offer little practical benefit). Another strategy, recommended by several participants, is the creation of networks or bodies to foster the development of research partnerships. However, while such high-level structures might facilitate matchmaking and ease some of the administrative burden on individuals and organizations who wish to partner, it is difficult to see how they would directly impact any of the identified barriers. Researchers and decision-makers, even if eager to participate in broad conversations, may continue to operate under different incentives, priorities, timelines and constraints. In contrast, the strategy of embedding researchers in organizations could eliminate many sources of misalignment between the two parties’ interests, thus removing multiple “researcher-side” barriers. Unfortunately, it does not address “decision-maker–side” barriers; even if embedded researchers provide exactly the knowledge requested, organizations may prove unwilling or unable to use it. (Organizations may also be unwilling to finance embedded research positions.)

It may be unrealistic to expect any strategy, or even a combination of strategies, to create a macro-context devoid of barriers to effective IKT. Many of the barriers are deeply entrenched, and some cannot or should not be eliminated. However, it might be possible to identify micro-contexts – configurations of issues, participants, settings, processes, and project characteristics – in which the approach seems most likely to achieve its goals, and try to create more of those contexts. Given increasing evidence that partnership poses significant costs to both researchers and decision-makers,^4^ it might also be desirable to identify unpromising contexts and discourage projects that are unlikely to succeed.

During my eight years as a member of an embedded research unit, one practice we found highly beneficial was to invest time in helping decision-makers pinpoint their knowledge needs. We used variants of the questions “What do you need to know in order to do your job?” and “What would you do with this information?” and gave feedback if we doubted that the requested knowledge would provide an appropriate basis for action. Not only did this process yield more useful research, evaluation, or knowledge-synthesis questions, it also helped us weed out questions that were unlikely to spark organizational action (eg, those posed by people with no intention and/or power to act on the issue) or unfeasible to answer in a timely and cost-effective manner. When we lacked the opportunity to help decision-makers refine their questions, or – as a result of senior management pressure or our own wishful thinking – proceeded despite red flags, the project tended to have lower impact. One strategy for promoting effective IKT might be to support this crucial phase, and demand evidence of its completion before allowing a proposal to move forward. This could assist researcher–decision-maker teams who are collaborating in good faith and help to dissuade those who are not. Many of the identified barriers should become apparent at this early stage (or, in the case of barriers to meaningful collaboration, even prior to it), allowing all parties to avoid low-yield endeavours.

In their thoughtful analysis of the difficulties of putting coproduction into practice, Bowen et al offer an encouraging sign that the literature is moving beyond the question of *whether* coproduction is beneficial to that of *how, for whom, and under what conditions* it can be so.^1,4,8,10^ Continued investigation of real-world experiences is needed to better understand the contexts and strategies that may best enable partnered research to fulfil its promise.

## Acknowledgements

I am grateful to Reena Kreindler for her editorial advice.

## Ethical issues

Not applicable.

## Competing interests

Author declares that she has no competing interests.

## Author’s contribution

SAK is the single author of the paper.
